# Virulence as a Side Effect of Interspecies Interaction in *Vibrio* Coral Pathogens

**DOI:** 10.1128/mBio.00201-20

**Published:** 2020-07-21

**Authors:** Esther Rubio-Portillo, Ana B. Martin-Cuadrado, Andrés M. Caraballo-Rodríguez, Forest Rohwer, Pieter C. Dorrestein, Josefa Antón

**Affiliations:** a Department of Physiology, Genetics and Microbiology, University of Alicante, Alicante, Spain; b Department of Biology, San Diego State University, San Diego, California, USA; c Skaggs School of Pharmacy and Pharmaceutical Sciences, University of California, San Diego, La Jolla, California, USA; d Viral Information Institute, San Diego State University, San Diego, California, USA; Max Planck Institute for Marine Microbiology

**Keywords:** *Vibrio*, virulence, competition, coral diseases, transcriptome

## Abstract

The increase in prevalence and severity of coral disease outbreaks produced by *Vibrio* pathogens, and related to global warming, has seriously impacted reef-building corals throughout the oceans. The coral Oculina patagonica has been used as a model system to study coral bleaching produced by *Vibrio* infection. Previous data demonstrated that when two coral pathogens (Vibrio coralliilyticus and Vibrio mediterranei) simultaneously infected the coral O. patagonica, their pathogenicity was greater than when each bacterium was infected separately. Here, to understand the mechanisms underlying this synergistic effect, transcriptomic analyses of monocultures and cocultures as well as experimental infection experiments were performed. Our results revealed that the interaction between the two vibrios under culture conditions overexpressed virulence factor genes (e.g., those encoding siderophores, the type VI secretion system, and toxins, among others). Moreover, under these conditions, vibrios were also more likely to form biofilms or become motile through induction of lateral flagella. All these changes that occur as a physiological response to the presence of a competing species could favor the colonization of the host when they are present in a mixed population. Additionally, during coral experimental infections, we showed that exposure of corals to molecules released during V. coralliilyticus and V. mediterranei coculture induced changes in the coral microbiome that favored damage to coral tissue and increased the production of lyso-platelet activating factor. Therefore, we propose that competition sensing, defined as the physiological response to detection of harm or to the presence of a competing *Vibrio* species, enhances the ability of *Vibrio* coral pathogens to invade their host and cause tissue necrosis.

## Abstract (precis)

Vibrio coralliilyticus and Vibrio mediterranei are important coral pathogens capable of inducing serious coral damage, which increases severely when they infect the host simultaneously. This has consequences related to the dispersion of these pathogens among different locations that could enhance deleterious effects on coral reefs. However, the mechanisms underlying this synergistic interaction are unknown. The work described here provides a new perspective on the complex interactions among these two *Vibrio* coral pathogens, suggesting that coral infection could be a collateral effect of interspecific competition. Major implications of this work are that (i) *Vibrio* virulence mechanisms are activated in the absence of the host as a response to interspecific competition and (ii) release of molecules by *Vibrio* coral pathogens produces changes in the coral microbiome that favor the pathogenic potential of the entire *Vibrio* community. Thus, our results highlight that social cues and competition sensing are crucial determinants of development of coral diseases.

## INTRODUCTION

The coral holobiont is a very rich consortium whose components interact in complex ways (1). Within natural environments, many taxa coexist in close proximity, and bacteria have evolved to sense and adapt to cues from neighboring species to enable them to communicate, cooperate, or compete (2). These species interactions define the pathogenic potential of the entire community (3, 4). Currently, it is well known that many coral diseases are caused by a diverse polymicrobial consortium (5–7). Two species in particular, Vibrio mediterranei (= Vibrio shilonii) (8) and Vibrio coralliilyticus, have caused coral deaths worldwide (9–15). Potential virulence factors have been identified by sequencing the genomes of these two *Vibrio* species (16–18) (and each of them has been shown to be virulent in laboratory coral infection assays [9, 10, 19, 20]). Although most infection experiments have studied these species separately, we recently demonstrated increased virulence and higher coral tissue damage under conditions in which V. coralliilyticus and V. mediterranei were inoculated simultaneously in Oculina patagonica, suggesting that the presence of both species resulted in a synergistic effect on pathogenic behavior (15). It was previously suggested that bacterial intraspecific competition can increase virulence (21), but that study did not present details about how virulence factors could be regulated by intraspecific competition. Thus, investigations of whether V. coralliilyticus and V. mediterranei interact synergistically may improve our understanding of coral diseases and facilitate the development of effective disease control strategies. Within this framework, the main goal of this study was to elucidate the mechanisms involved in pathogenic synergy and virulence factor regulation in response to the presence of another pathogen. Transcriptional responses were evaluated at two temperatures under monoculture and coculture conditions. These experiments showed that these two bacteria increased production of multiple virulence factors during coculture (in the absence of corals) in a temperature-dependent matter specific to each species. These findings highlight that competition sensing, defined as a physiological response to detection of harm or to the presence of a competing *Vibrio* species, increased the pathogenic potential in these two coral pathogens, which could favor host invasion. Additionally, we showed that metabolites released by these two coral pathogens influenced the coral microbial community, favoring the growth of other potential pathogens and subsequent coral tissue necrosis.

## RESULTS AND DISCUSSION

### Growth and cocultivation.

The growth of the two vibrios was monitored in monoculture and coculture by DAPI (4′,6-diamidino-2-phenylindole) and FISH (fluorescence *in situ* hybridization) counts, respectively (see Fig. S1 in the supplemental material). Growth rates were similar for monocultures of the two species, although V. mediterranei grew slightly faster than V. coralliilyticus at 20°C whereas V. coralliilyticus grew faster than V. mediterranei at 28°C. Although both species grew slower in coculture, the growth of V. mediterranei was less influenced by cocultivation at 20°C and V. coralliilyticus was less affected at 28°C. Therefore, growth data suggest that V. mediterranei is more adapted to 20°C and V. coralliilyticus to 28°C.

10.1128/mBio.00201-20.1 FIG S1 Growth rates of Vibrio coralliilyticus and Vibrio mediterranei in monoculture and coculture at 20 and 28°C. The growth curves were prepared using cell counts with DAPI in monocultures and FISH in cocultures performed each hour for 11 h. Error bars represent standard deviations of results from three replicate samples. Download FIG S1, TIF file, 0.06 MB. Copyright © 2020 Rubio-Portillo et al. 2020 Rubio-Portillo et al. This content is distributed under the terms of the Creative Commons Attribution 4.0 International license.

### Transcriptomic analysis under coculture conditions.

**FIG 1:**
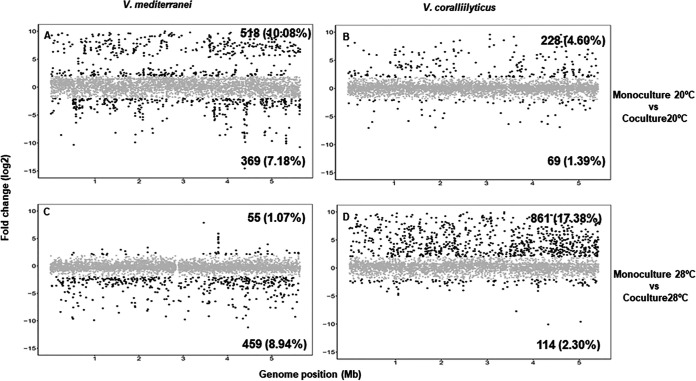
Fold changes across the genomes of V. mediterranei and V. coralliilyticus. The plots show comparisons between coculture and monoculture conditions at 20°C (A and B) and 28°C (C and D). In each plot, the genes that have a fold change (log_2_) of greater than 2 or less than −2 in the genomes of V. mediterranei (A and C) and V. coralliilyticus (B and D) are colored in black. Genes represented in gray are those with fold change values between −2 and 2. The number of genes upregulated (upper right corner in each plot) or downregulated (bottom right corner in each plot) and the percentage of total genes affected are shown in bold.

During exponential growth of each culture, RNA was extracted and sequenced for transcriptome analysis by comparison of the genomes of V. coralliilyticus and V. mediterranei, which were sequenced and analyzed as described above. The main features of these genomes are shown in Table S2 in the supplemental material. Transcriptome sequencing (RNA-seq) data from replicates from the same condition were highly reproducible, showing Pearson coefficient values between 0.977 and 0.981. Between 1.29 × 10^6^ and 9.08 × 10^6^ forward reads were obtained for each of the eight samples, and the total number was reduced to between 4.5 × 10^5^ and 3.84 × 10^6^ mRNA reads after excluding reads mapping to rRNA genes (Table S3). The percentage of expressed open reading frames (ORFs) depended on the growth conditions, with significantly fewer ORFs expressed under coculture conditions at 20°C and 28°C for V. coralliilyticus and V. mediterranei, respectively (Table S4), which was consistent with the growth rate data shown before. A comparison of gene expression levels clearly showed that, for the two strains, cocultures had significantly more overexpressed genes at 20°C and 28°C for V. mediterranei and V. coralliilyticus, respectively (Fig. 1). More specifically, for V. mediterranei at 20°C, 887 genes were differentially expressed in coculture compared with monoculture (Fig. 1A). Among these, 518 genes were upregulated and most of them were related to environmental adaptation, cell motility, signaling molecules and interactions, drug resistance, virulence, and enzyme synthesis (Fig. S2A). In V. coralliilyticus, 975 genes were differentially expressed in coculture compared with monoculture at 28°C and 861 of these were upregulated (Fig. 1D). Most of those genes with known function were classified as metabolic genes, but approximately 20% were related to infection diseases, enzyme synthesis, cellular community, cell motility, and drug resistance (Fig. S2D). In summary, the results showed that a great proportion of the upregulated genes under coculture conditions were related to competition sensing and virulence. Among them, genes of known functional and pathogenic significance were selected and further analyzed as described below.

10.1128/mBio.00201-20.2 FIG S2 KEGG pathway classifications of Vibrio coralliilyticus and Vibrio mediterranei genes. The horizontal axis shows the KEGG categories. The vertical axis displays the percentages of annotated genes whose expression was upregulated (yellow), downregulated (red), or unchanged (gray) in Vibrio mediterranei under coculture conditions at 20°C (A) and 28°C (B) and in Vibrio coralliilyticus under coculture conditions at 20°C (C) and 28°C (D). Download FIG S2, TIF file, 2.6 MB. Copyright © 2020 Rubio-Portillo et al. 2020 Rubio-Portillo et al. This content is distributed under the terms of the Creative Commons Attribution 4.0 International license.

### Virulence-related genes overexpressed in cocultures.

**FIG 2:**
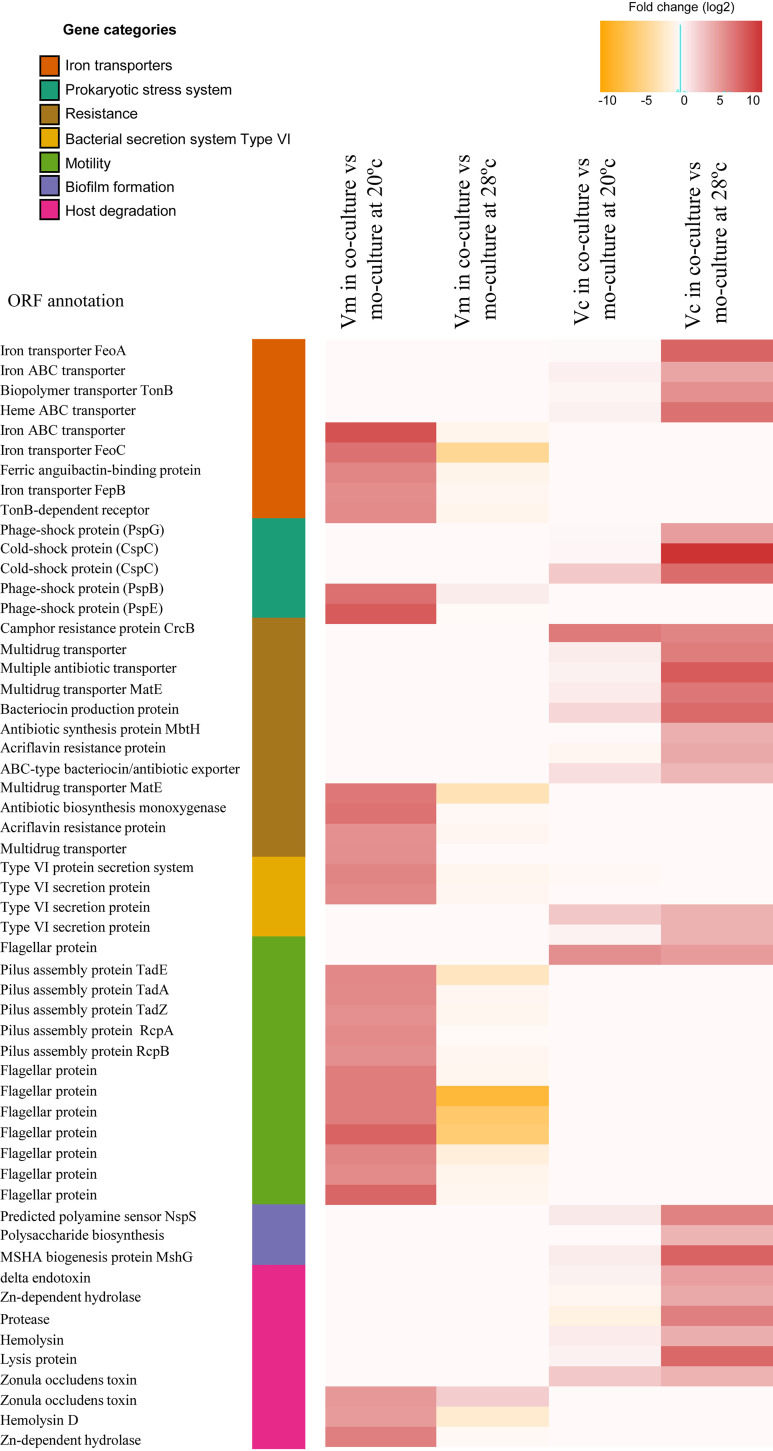
Heat map visualization of Vibrio mediterranei (Vm) and Vibrio coralliilyticus (Vc) genes that showed more highly differential expression levels under monoculture and coculture conditions. Each row represents a gene (ordered by categories), and each cell represents the fold change detected between the conditions.

**FIG 3:**
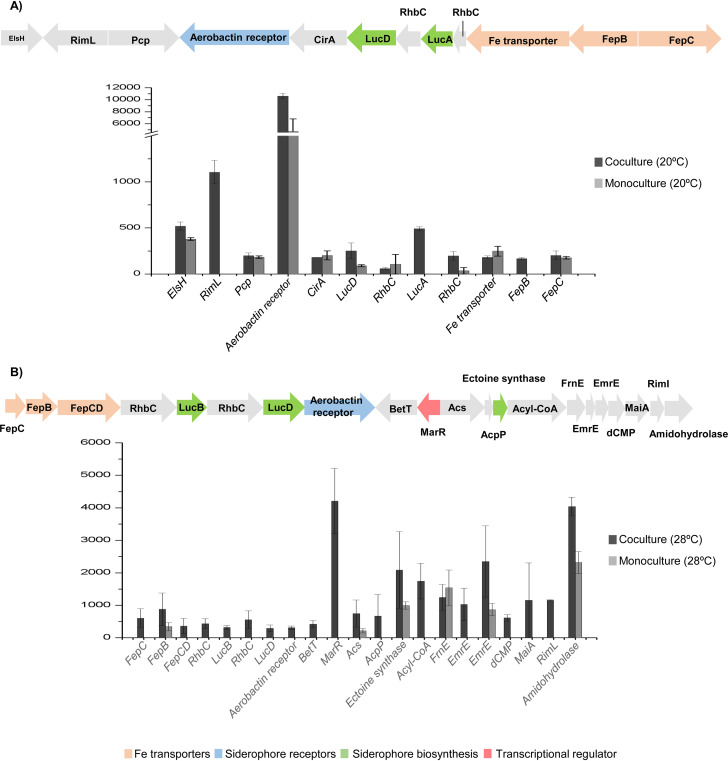
Expression levels of siderophore biosynthesis genes under *Vibrio* coculture conditions. (A) Siderophore biosynthesis cluster of Vibrio mediterranei and the reads per kilo base per million reads (RPKM) values of each gene expression measured at 20°C in monoculture and coculture conditions. (B) Siderophore biosynthesis cluster of Vibrio coralliilyticus and the reads per kilo base per million reads (RPKM) values of each gene expression measured at 28°C in monoculture and coculture conditions. Acyl-CoA, acyl coenzyme A.

**(i) The siderophore systems.** Genes encoding iron transporters were overexpressed under coculture conditions (Fig. 2). Using antiSMASH (22), we identified aerobactin siderophore biosynthetic clusters in chromosome II from both *Vibrio* spp. In V. mediterranei, this cluster included 12 ORFs, which were overexpressed in coculture at 20°C but not at 28°C. Conversely, in V. coralliilyticus, this cluster (with 10 ORFs) was highly upregulated under coculture conditions at 28°C (Fig. 3). These differences were not due to different iron concentrations under monoculture and coculture conditions, as chemical analysis indicated that the iron concentrations were similar under both conditions (Fig. S3). It can be concluded that siderophores may have been produced because of the presence of the other *Vibrio* spp. in the cocultures in a manner similar to that previously detected for an increase of siderophore production as a response to competitors in Pseudomonas aeruginosa (23, 24). In addition to iron acquisition, siderophores in pathogenic vibrios are also involved in production of several virulence factors such as toxins (25), biofilm formation (26), and swarming motility (27). Thus, *Vibrio* coral pathogens that produce siderophores in complex microbial communities may have a selective advantage through their ability to obtain iron that is not freely available in the coral host such as has been described previously in other *Vibrio* species (28). This production could also increase *Vibrio* virulence through, for instance, toxin production (see below).

10.1128/mBio.00201-20.3 FIG S3 Extracellular iron concentrations in monoculture and coculture conditions at 20°C (gray bars) and 28°C (black bars). Inductively coupled plasma mass spectrometry (ICP-MS) was used for the measurements, and iron concentrations are displayed in parts per billion (ppb). Three independent replicates were performed, and mean values ± standard deviations (SD) (error bars) are displayed in each bar. Download FIG S3, TIF file, 0.3 MB. Copyright © 2020 Rubio-Portillo et al. 2020 Rubio-Portillo et al. This content is distributed under the terms of the Creative Commons Attribution 4.0 International license.

**(ii) Type VI secretion systems.** Bacteria have a variety of specialized secretion systems that enable cells to respond to the environment through adhesion, pathogenicity, adaptation, and competition (29). The type VI secretion system (T6SS) proteins function as virulence factors against eukaryotic and prokaryotic cells and play an important role during antagonistic interactions with competing microorganisms (30). In Vibrio cholerae, the T6SS is a contact-dependent weapon that enables direct killing of other cells by the translocation of proteinaceous toxins into its competitors. Therefore, this system plays an important role during antagonistic interactions with competing microorganisms, promoting V. cholerae survival in the aquatic lifestyle and in human hosts (31). In our experiments, genes encoding T6SS-related proteins were clearly upregulated in both *Vibrio* species under coculture conditions (fold change > 4; Fig. 2). V. mediterranei genes related to the T6SS were upregulated in cocultures at 20°C, while they were upregulated in V. coralliilyticus at 28°C. Therefore, the activation of T6SS as a competition sensing mechanism in *Vibrio* cocultures was able to promote interspecies toxicity and to favor V. mediterranei and V. coralliilyticus survival against competing microorganisms during coral colonization at 20 and 28°C, respectively.

**(iii) Prokaryotic stress systems.** Typically, stress responses are initiated to enable cells to adapt to a new environment, such as a change in nutrient availability, species competition, or attack from the host immune system. For this reason, stress responses could be considered a mechanism to detect ecological competition from other cells that are present (32). A common strategy followed by several microbes in this ecological competition is that of developing antibiotic resistance as a response to the threat of toxin attack by other cells (32). In our experiments, genes related to efflux pumps and drug resistance were more highly expressed under coculture conditions than under monoculture conditions (Fig. 2), suggesting that the products of these genes could mediate competition between these species. In addition to antibiotic resistance, we detected upregulation of proteins related to other stress responses in cocultures. V. mediterranei overexpressed genes related to phage shock proteins (Psp) at 20°C (Fig. 2), while genes encoding cold shock proteins (Csp) were upregulated in V. coralliilyticus at 28°C. The Psp response induces synthesis of essential mechanisms for virulence in many pathogens (33, 34), while Csp-like proteins play an important role in survival during host colonization (35). Consequently, when these two *Vibrio* species cooccur in the coral host, activating stress responses could favor V. mediterranei and V. coralliilyticus survival during coral colonization at 20 and 28°C, respectively.

**(iv) Motility.** *Vibrio* species have a dual flagellar system that includes a constitutive polar flagellum required for swimming motility and a lateral flagellar system related to swarming motility that is induced only under certain conditions and is essential for movement in viscous environments (36–38). According to our findings, the lateral flagellar locus in V. mediterranei was overexpressed under coculture conditions at 20°C, together with genes that encode the tight adherence pili (Fig. 2), which are essential for adherence, biofilm formation, colonization, and pathogenesis (39). Similarly, several genes involved in biogenesis of the type IV mannose-sensitive hemagglutinin (MSHA) pilus were upregulated in V. coralliilyticus under coculture conditions at 28°C (Fig. 2). Overexpression of MSHA genes was previously detected in heat-activated V. coralliilyticus transcriptomes (18), and these genes were also previously found to be involved in biofilm formation (40). Therefore, the interaction between V. mediterranei and V. coralliilyticus coral pathogens may induce changes in motility and biofilm formation that could favor coral colonization.

**(v) Production of lytic enzymes and toxins.** Pathogenic bacteria produce different substances that are directly or indirectly toxic to host cells; for example, lytic enzymes that cause damage to host tissues are known to play a central role in pathogenesis (41). Our results show that both species produced hemolysins and Zn-dependent metalloproteases in cocultures and that V. coralliilyticus also produced proteases (Fig. 2). It has been reported previously that Zn metalloproteases produced by V. mediterranei and V. coralliilyticus cause photosynthetic inhibition of the coral dinoflagellate endosymbiont *Symbiodinium* (87). In addition to lytic enzymes, these two *Vibrio* species in coculture also overexpressed the gene that encodes the zonula occludens toxin (Zot) (Fig. 2). In V. cholerae, this phage-encoded toxin increases the permeability of the human intestinal epithelium through reorganization of the cytoskeleton filaments by actin polymerization (42) and may have the same effect in the coral epithelial cell barrier (Rubio-Portillo et al., unpublished results).

**(vi) Noncoding RNAs (ncRNAs) as virulence regulators.** ncRNAs are small RNA transcripts (typically 50 to 250 nucleotides) that are not translated into proteins but instead play important roles as posttranscriptional regulators involved in diverse bacterial processes that include stress responses and pathogenicity (43, 44). Among the transcript families identified here, CsrB/RsmB and PrrB/RsmZ RNA had the highest overexpression in both species (Fig. S4). CsrB/RsmB ncRNAs participate in several global regulatory circuits such as cell motility, quorum sensing, and pathogenesis (45–47). RNAs within the PrrB/RsmZ family are structurally similar to CsrB/RsmB and might have similar roles (48). Overall, our results suggest that CsrB and (to a lesser extent) PrrB may be master regulators of the synergistic pathogenicity when the two vibrios grow together. This fits with the observed increased transcription of genes involved in toxin production and expression of flagellar genes, together with the observed rise in levels of cell density (quorum)-sensing signals or their associated pathogenic machinery. Because ncRNAs are not translated and thus are immediately available, they might rapidly modulate a wide range of cellular responses, which would allow *Vibrio* spp. to sense and rapidly respond to putative competitors or environmental stimuli.

10.1128/mBio.00201-20.4 FIG S4 Number of reads containing noncoding RNA (ncRNA) in *Vibrio* transcriptomes. The more abundant ncRNAs are compared across all conditions. Download FIG S4, TIF file, 0.9 MB. Copyright © 2020 Rubio-Portillo et al. 2020 Rubio-Portillo et al. This content is distributed under the terms of the Creative Commons Attribution 4.0 International license.

In summary, transcriptional data show that V. mediterranei and V. coralliilyticus were able to detect competitor cells and respond to ecological competition by producing siderophores for survival in the coral host, producing virulence factors (T6SS, toxins) against the host and associated microorganisms and expressing swarming motility and forming biofilms for host adherence. The activation of these mechanisms as a consequence of interspecific competition might favor *Vibrio* pathogenicity. Interestingly, the transcriptional responses to the interactions at 20 and 28°C were different for each species; whereas V. mediterranei displayed higher pathogenic potential at 20°C, V. coralliilyticus seemed to be more virulent at 28°C. These results agree with our previous results that showed that V. mediterranei and V. coralliilyticus induced more signs of disease in corals at lower temperature in cocultures than in the natural environment (15). Taken together, these results highlight the harmful potential of coral pathogens when they coexist in the same host. This raises concerns about the possible deleterious effects of the dispersal of pathogens among different locations and their consequences for coral health worldwide.

### O. patagonica infection experiments.

**FIG 4:**
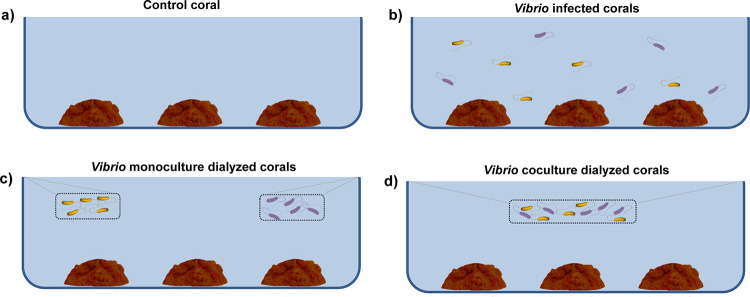
Coral infection experiment working scheme. (a) Control coral tank. (b) Tank with *Vibrio*-infected corals. (c) Tank with *Vibrio* monoculture dialyzed corals. (d) Tank with *Vibrio* coculture dialyzed corals.

Considering that our transcriptomic results highlighted the importance of interactions for the pathogenic potential of *Vibrio* and that corals species harbor a natural *Vibrio* community, we also attempted to determine how molecules released by *Vibrio* pathogens would affect *Vibrio* coral assemblages and coral health. With this purpose, laboratory infection experiments were conducted at two different temperatures (20 and 28°C). In these experiments, to distinguish between the effects of direct contact between *Vibrio* cells and coral and the effects of molecules released by *Vibrio* cells, corals were inoculated with the *Vibrio* coculture in either of two ways: cells were either free to interact with the coral or were contained inside a dialysis membrane. Thus, together with corresponding (uninfected) controls, three different treatments were set for each temperature: an infected tank where cells were free, a *Vibrio* coculture dialyzed tank, and a *Vibrio* monoculture dialyzed tank (Fig. 4). The health of the coral fragments used in the experiments was assessed by measurements of concentrations of chlorophyll *a* (Chl *a*) in their tissues. *Vibrio* cells in coral tissues were quantified by diluting and plating crushed coral tissue and counting the resulting *Vibrio* colonies.

### Effects on coral health status.

**FIG 5:**
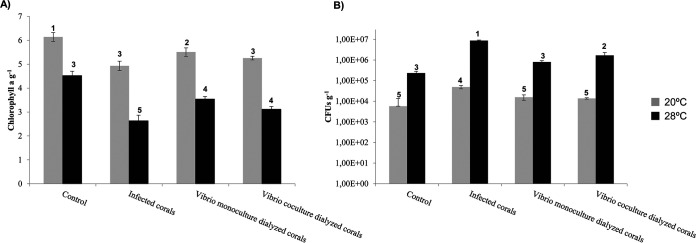
Levels of chlorophyll *a* (A) and concentrations of culturable *Vibrio* species (B) in O. patagonica samples after 10 days of experimental infection with the indicated treatment at two different temperatures (20°C and 28°C, shown with gray and black bars, respectively). Groups were defined by *post hoc* Tukey test after ANOVA within each locality and are indicated by numbers at the top of the columns.

Results showed that the chlorophyll *a* concentrations decreased significantly with temperature in all treatments (analysis of variance [ANOVA] *P* < 0.001) (Fig. 5A) and that increases in *Vibrio* abundances were detected concurrently (ANOVA *P* < 0.001) (Fig. 5B). Corals infected with *Vibrio* and corals in the presence of *Vibrio* cocultures inside dialysis membranes showed a decrease in chlorophyll *a* concentrations in their tissues compared to controls, independently of seawater (SW) temperature (Fig. 5A). Therefore, vibrios inside the membranes were likely producing molecules that affected the coral *Vibrio* community and coral health status. Nevertheless, the concentration of *Vibrio* in their tissues was statistically significant only at 28°C (Fig. 5B).

**FIG 6:**
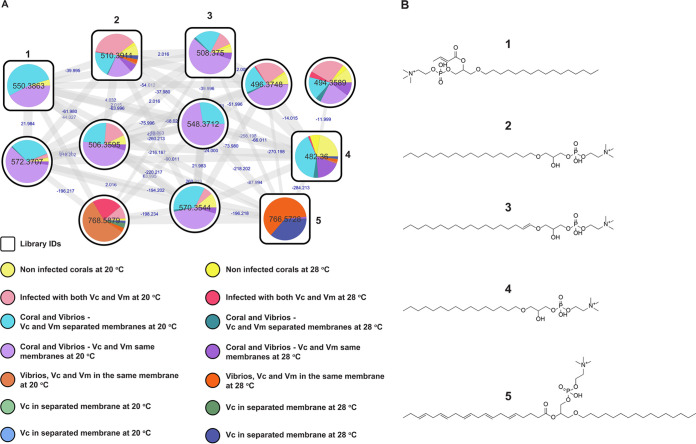
Phospholipid molecular family (platelet activator factor-related molecules, PAFs) from Oculina patagonica exposed to Vibrio coralliilyticus and Vibrio mediterranei. (A) Molecular family showing the relative abundance of detected phospholipids as pie charts. Labeled nodes correspond to the chemical structures in panel B. Each node represents a detected molecular feature. Edges connecting nodes represent spectral similarity, creating subnetworks (molecular families). Edge labels between the connected nodes represent the mass shift between related detected features. Color codes and legends included in the figure correspond to the experimental setup described in the Fig. 4 legend. (B) Chemical structures of putatively identified phospholipids are indicated as follows: 1, feature *m/z* 550.3863, annotated as 1-O-hexadecyl-2-O-(2E-butenoyl)-sn-glyceryl-3-phosphocholine; 2, feature *m/z* 510.3911, annotated as 1-octadecyl-sn-glycero-3-phosphocholine (Lyso PC O-18:0); 3, feature *m/z* 508.3750, annotated as 1-(1Z-octadecenyl)-sn-glycero-3-phosphocholine; 4, feature *m/z* 482.3600, annotated as 1-O-hexadecyl-sn-glycero-3-phosphocholine (Lyso-PAF); 5, feature *m/z* 766.5728, annotated as eicosapentaenoyl PAF C-16. Similarity to annotated fragmentation spectra from the GNPS repository and calculated molecular formula based on detected accurate mass were used to identify compounds. These identifications correspond to level 2 for compounds 2 and 4 and level 3 for compounds 1, 3, and 5, as stereochemistry was not established. The levels of identification correspond to suggested annotations according to metabolomics standards (83, 84). IDs, identifiers.

Metabolome analysis showed a high abundance of platelet activating factors (PAF), such as Lyso-PAF-C:16, in O. patagonica tissues exposed to vibrios (Fig. 6A and B). Activation of Lyso-PAFs has been seen previously in terrestrial invertebrates in response to tissue damage (49), as well as in corals (50–52). Lyso-PAFs seem to be an important element of the immune responses of basal metazoans (51) and have antimicrobial properties (53). The levels of this class of phospholipids were measured for all coral samples, and their relative abundances were higher in infected corals and corals in the presence of *Vibrio*s than in the control corals. Curiously, the abundance of Lyso-PAF-C:16 was higher in coral tissues when O. patagonica was exposed to *Vibrio* strains inside dialysis membranes at 20°C (Fig. S5). This fact supports the hypothesis that vibrios produce signaling molecules that induce changes in the coral holobiont and its microbial communities and ultimately affect their health status.

10.1128/mBio.00201-20.5 FIG S5 One-way ANOVA (Kruskal-Wallis test; adjusted *P* value [false-discovery-rate {FDR} cutoff, 0.05]) for detected features 1, 2, 3, 4, and 5 as shown in Fig. 6. Feature 5 (PAF C-16) was more abundant when Oculina patagonica was inoculated with both *Vibrio* strains at 28°C. Feature 2 (Lyso PC O-18:0) was more abundant in the presence of both *Vibrio* strains at 20°C. The color code does not correspond to the colors used in panel A. Legends included in the figure correspond to the following: 20C (representing noninfected coral at 20°C); 28C (noninfected coral at 28°C); 20I (infected with both V. coralliilyticus and V. mediterranei at 20°C); 28I (infected with both V. coralliilyticus and V. mediterranei at 28°C); 20MS (coral and vibrios V. coralliilyticus and V. mediterranei in separated membranes at 20°C); 28MS (coral and vibrios V. coralliilyticus and V. mediterranei in separated membranes at 28°C); 20MX (coral and vibrios V. coralliilyticus and V. mediterranei in the same membrane at 20°C); 28MX (coral and vibrios V. coralliilyticus and V. mediterranei in the same membrane at 28°C); MemMix20 (vibrios V. coralliilyticus and V. mediterranei in the same membrane at 20°C); MemMix28 (Vibrios V. coralliilyticus and V. mediterranei in the same membrane at 28°C); MemV. coralliilyticus20 (V. coralliilyticus in a separated membrane at 20°C); MemV. coralliilyticus28 (V. coralliilyticus in a separated membrane at 28°C); MemV. mediterranei20 (V. mediterranei in a separated membrane at 20°C); MemV. mediterranei28 (V. mediterranei in a separated membrane at 28°C). Statistical analyses were performed using the MetaboAnalyst platform (85–87). Download FIG S5, TIF file, 3.2 MB. Copyright © 2020 Rubio-Portillo et al. 2020 Rubio-Portillo et al. This content is distributed under the terms of the Creative Commons Attribution 4.0 International license.

### Changes in coral *Vibrio* assemblages induced by exposure to V. mediterranei *and* V. coralliilyticus.

**FIG 7:**
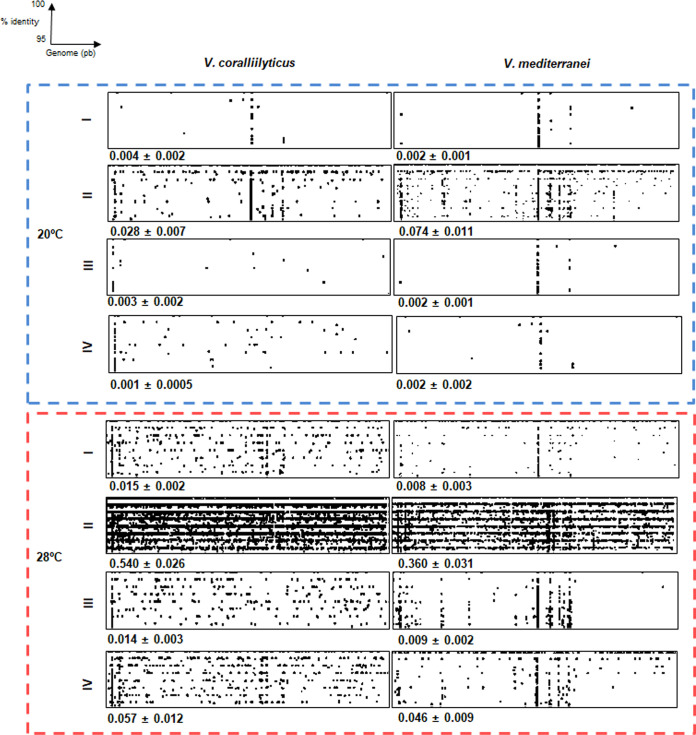
Fragment recruitment plots for metagenome sequence reads of Vibrio coralliilyticus and Vibrio mediterranei genomes from each coral treatment (treatment I, control corals; II, infected corals; III, corals in the presence of *Vibrio* monocultures; IV, corals in the presence of *Vibrio* coculture). The vertical axis indicates the sequence identity of an alignment between a metagenomic sequence and the reference *Vibrio* genome determined using BLASTn; the levels of identity range from 100% (top) to 95% (bottom). The percentage of reads belonging to each *Vibrio* species is shown in bold in the corner of each plot. Genome (pb), genomes quantified as base pairs.

To assess the abundances of the two *Vibrio* coral pathogens in the coral microbiome, we sequenced the metagenomes of corals used in these experiments and performed fragment recruitment analysis. This analysis indicated that both species were more abundant in corals maintained at 28°C, including the untreated controls (Fig. 7). These results are consistent with our previous findings (54), confirming that these *Vibrio* species form part of the natural coral microbiota and that they increase their abundances in response to elevated seawater temperature. We detected an increase in the levels of these *Vibrio* coral pathogens in coral tissues in directly coinfected corals compared to control corals, which suggests that these pathogens were actively infecting coral tissues. V. mediterranei was more abundant in corals maintained at 20°C, while V. coralliilyticus was more abundant at 28°C (Fig. 7). This result, together with the results obtained by transcriptomic analysis and our previous finding that V. mediterranei is more virulent at 20°C and V. coralliilyticus is more virulent at 28°C (15), suggests that V. mediterranei is more highly adapted to low temperatures than V. coralliilyticus.

**FIG 8:**
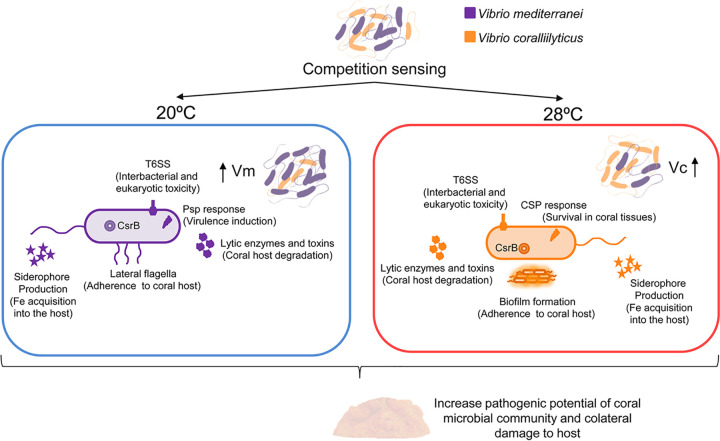
Model depicting how *Vibrio* competition triggers their virulence over coral hosts. We propose that the synergism observed in coinoculated corals was caused by increases in *Vibrio* virulence as a consequence of competition sensing mechanisms. *Vibrio* coral pathogens within corals exist in close proximity with many taxa, and their interactions define the pathogenic potential of the entire community. *Vibrio* spp. can respond to competition by attacking via the T6SS or by defending themselves through induction of stress responses. In addition, *Vibrio* spp. produce siderophores that are involved in biofilm formation, swarming motility, and toxin production. These mechanisms, activated by competition sensing, may confer an advantage to *Vibrio* coral pathogens that enables them to survive in host tissue and degrade the tissue as collateral damage.

An in-depth analysis of the presence or absence of other *Vibrio* species using fragment recruitment-based abundances in coral metagenomes showed that the abundances of Vibrio splendidus and Vibrio atlanticus, previously reported to be part of the “constitutive” O. patagonica microbiota (15), decreased at 28°C and also in infected corals and corals exposed to *Vibrio* coral pathogens in dialysis at 20°C (Fig. S6). In contrast, the *Vibrio* species that are potential coral pathogens, such as Vibrio harveyi or Vibrio alginolyticus (55, 56), had increased relative abundances at 28°C and their abundances were even higher in corals that were infected or were maintained in the presence of V. mediterranei and V. coralliilyticus (Fig. 8). Other example was the well-known human-pathogenic species Vibrio parahaemolyticus, which can also cause infections in aquatic organisms (57); V. parahaemolyticus also had an increased relative abundance in the presence of V. mediterranei and V. coralliilyticus (Fig. 8). Summarizing, the presence of V. coralliilyticus and V. mediterranei not only changed their own relative abundances in corals but also altered the coral microbiome, promoting the growth of other potential *Vibrio* coral pathogens.

10.1128/mBio.00201-20.6 FIG S6 Fragment recruitment plots for metagenome sequence reads on seven reference *Vibrio* genomes (see Table S1) from each coral treatment (I, control corals; II, infected corals; III, corals in the presence of *Vibrio* monocultures; IV, corals in the presence of *Vibrio* coculture). The vertical axis indicates the sequence identity of an alignment between a metagenomic sequence and the reference *Vibrio* genome using BLASTn. Identity ranges from 100% (top) to 95% (bottom). The percentage of reads belonging to each *Vibrio* species is shown at the bottom of each plot. Download FIG S6, TIF file, 2.9 MB. Copyright © 2020 Rubio-Portillo et al. 2020 Rubio-Portillo et al. This content is distributed under the terms of the Creative Commons Attribution 4.0 International license.

### Conclusions.

V. mediterranei and V. coralliilyticus, which are involved in coral diseases, display synergetic interactions that resulted in enhanced pathogenesis in coinfection experiments (15). Our results provide evidence that these *Vibrio* coral pathogens activate mechanisms that they use to invade and cause harm to their coral hosts in the absence of coral and thus may also use these mechanisms to attack competing species in mixed populations. It is noteworthy that these coral-damaging weapons are expressed in the absence of the host and thus seem to be targeting the competing bacteria rather than the infected host. Therefore, we propose that the synergism observed in coinfected corals was caused by increased virulence as a side effect of interspecies competition (Fig. 8). Furthermore, we also demonstrated that molecules released by these two *Vibrio* pathogens produced changes in the coral microbiome which enhanced the pathogenic potential of the entire community by increasing abundances of potential coral pathogens and therefore coral disease susceptibility.

## MATERIALS AND METHODS

### Experiments *in vitro*.

**(i) Strain cultivation, monocultures, and cocultures.** V. mediterranei (strain Vic-Oc-097; CECT 30098) and V. coralliilyticus (strain Vic-Oc-068; CECT 30097) strains, originally isolated from a bleached colony of O. patagonica (15), were used in this work. Triplicate monocultures and cocultures were set by inoculating 1 × 10^9^ cells, determined by DAPI (4′,6-diamidino-2-phenylindole) staining, into 250 ml LB medium with 3% NaCl and incubating at either 20 or 28°C. Growth was monitored by optical density at 600 nm (OD_600_) and DAPI staining. Relative amounts of V. mediterranei and V. coralliilyticus cells in cocultures were determined by fluorescence *in situ* hybridization (FISH) with species-specific probes (see below). These probes were also used to check for cross-contamination in monocultures. The growth curves for each condition were used to establish the middle of exponential-growth phase, which was the time point for transcriptome analyses.

**(ii) FISH with species-specific probes.** V. mediterranei-specific and V. coralliilyticus-specific probes targeting 16S rRNA were designed with the probe-designing tool of the ARB software package. Based on comparative analysis of all sequences in the Living Tree Project database (LTP project [58]; http://www.arb-silva.de/projects/living-tree) and our *Vibrio* genomic sequences, the program selected specific regions to discriminate between the two species. The oligonucleotide probes generated were ViVc-FISH (5′-TCGTTCCCCTAAGGTTCAGACA-3′) and ViVm-FISH (5′-CAGTCACCCGAAGGGTCAGTTA-3′) for V. coralliilyticus and V. mediterranei, respectively, and were labeled with Cy-5. Cells were fixed in 4% formaldehyde for 12 h at 4°C and then washed with phosphate-buffered saline (PBS) and kept at 4°C until their analysis. Hybridizations were performed at 46°C for 5 h with hybridization buffer (900 mM NaCl, 20 mM Tris-HCl [pH 8], 0.01% sodium dodecyl sulfate) containing 2 ng of labeled probe/μl. The hybridization stringency was adjusted by adding different formamide concentrations (from 10% to 70% [vol/vol] with increments of 10%) to the hybridization buffer (best results were obtained using 40% for ViVc-FISH and 35% for ViVm-FISH). Hybridizations were washed at 48°C for 30 min with washing buffer containing the same components as the hybridization buffer except for the probes and formamide.

**(iii) Whole-genome sequencing.** Total bacterial DNA was extracted using a DNeasy blood and tissue kit (Qiagen, Valencia, CA) and sequenced using an Illumina MiSeq platform to generate 2 × 300-bp reads according to the manufacturer’s protocol. Raw reads were cleaned up by trimming the adaptor sequences and low-quality ends (quality score < 30) using Trimmomatic (59). The cleaned reads were *de novo* assembled using the SPAdes Genome Assembler (60). ORF detection was performed with Prodigal (61). Functional annotation was performed by comparing predicted protein sequences through BLAST comparisons against the NCBI-nr database, Pfam (62), COG (63), and TIGRFAM (64) (cutoff E value, 10^−5^). Ribosomal genes were identified using RNAscan (65). BLAST average nucleotide identity (ANIb) values were calculated based on the whole-genome sequence as described previously by Goris et al. (66). Analysis of the genome completeness was performed using CheckM (67). tRNAscan was used to predict the presence of tRNAs in the genomes as described previously by Lowe and Chan (68). Synteny among the V. mediterranei and V. coralliilyticus genomes and those of other vibrios was analyzed using the Artemis Comparison Tool (ACT) from Artemis package v.18.0.2 (69). Finally, GC content data were calculated using the “geecee” tool from the emboss package (70).

**(iv) RNA extraction and sequencing.** Two replicate aliquots from cultures maintained under each set of conditions were centrifuged at 16,000 × *g* for 15 min, and the resulting cell pellets were preserved at –80°C until RNA was extracted. Total RNA was extracted using an RNeasy minikit (Qiagen) in accordance with the instructions from the manufacturer. Genomic DNA was removed from the extracted RNA by treating the samples with Turbo DNase (Ambion) at 37°C for 45 min. rRNA was partially eliminated with a Ribo-Zero rRNA removal kit (Epicentre), and samples were sequenced using 150-bp paired-end reads on an Illumina NextSeq500 platform.

**(v) Analysis of gene expression and detection of differentially expressed genes.** Raw reads were cleaned by trimming the adaptor sequences and low-quality ends (quality score < 30) using Prinseq (71). Forward reads were mapped against *Vibrio* genomes using bowtie 2 (72). Once the forward reads from monocultures were mapped to each genome, the reads from cocultures were aligned against a combined reference containing the genomes of V. mediterranei and V. coralliilyticus. The expression profiles for monocultures were normalized using the number of cells and the ORF length, and these values were provided as RPKM (reads per kilobase per million). Similarly, cocultures were also normalized by the number of cells of each *Vibrio* species. Differentially expressed genes, defined as genes whose expression levels differed more than 2-fold between the two conditions, were identified using DEseq, a variance analysis package that was developed to infer statistically significant differences in gene expression data from high-throughput sequencing (73).

**(vi) Analysis of noncoding RNA (ncRNA).** We used the cmsearch program as described previously by Cui et al. (74) and the Rfam database (75) to screen for the presence of ncRNAs among the short reads of the transcriptome collections. For this purpose, the remaining ribosomal genes (the 16S, 23S, and 5S rRNA genes) were first removed from the data set using RNAmmer (76). A subset of 10^5^ reads was extracted randomly for each of the samples and submitted to the ncRNA screening. For stringency, an E value of 10^−5^ was used as the cutoff.

### O. patagonica infection experiments.

Fragments (about 5 cm of diameter) of the coral O. patagonica were collected in June 2016 (seawater from the Marine Reserve of Tabarca, Spain) and transferred to the laboratory. They were acclimated for 3 days in aquaria (20 liters) and then were randomly assigned to 1 of 8 aquaria (3 liters, 3 pseudoreplicated colonies per aquarium). Afterward, fragments were slowly acclimated to the experimental temperature by increasing the temperature by 0.5°C per day from 18°C (seawater temperature at sampling location) until the final temperature was reached (20 or 28°C). Before inoculation, the corals were maintained at the experimental temperature for 3 days. Water was replaced every 3 days during the 10 days of the infection experiment. In order to study non-contact-dependent effects of *Vibrio* pathogens on coral, a series of infections using dialysis membranes were included in the experiment. These membranes prevented contact between the coral and the *Vibrio* cells but allowed the free dilution of molecules with a molecular weight lower than 100 kDa. One set of four tanks was incubated at 20°C and another set at 28°C. Each set of tanks contained the following: (I) control corals with no *Vibrio* added; (II) corals inoculated with the *Vibrio* coculture; (III) corals with a *Vibrio* coculture growing inside a dialysis membrane; and (IV) corals in the presence of the two *Vibrio* monocultures, each growing independently inside a dialysis membrane (Fig. 4). Aquaria were inoculated to reach a final bacterial concentration of 10^3^ cells/ml of seawater, and the same *Vibrio* concentration was added into the dialysis membranes. At the end of the experiments, coral colonies were gently washed three times with 50 ml of sterile filtered seawater (SFSW) to remove nonassociated bacteria, broken into small pieces, placed in 50-ml tubes, and centrifuged for 3 min at 2,900 × *g* (Labofuge 400R; Heraeus Instruments) to separate the mucus from the coral tissue and skeleton. After centrifugation, the coral pieces were crushed in SFSW using a mortar and pestle, and the CaCO_3_ skeleton was allowed to settle for 15 min before the supernatant (i.e., the crushed tissue) was collected and used for *Vibrio* counts, measurements of chlorophyll *a* (Chl *a*) concentrations, and extractions of DNA and metabolites.

For plate counts of *Vibrio* spp., 10-fold serial dilutions of crushed tissue were prepared in SFSW, plated on thiosulphate citrate bile sucrose (TCBS) agar (Pronadisa, Spain), and incubated at 30°C for 48 h. For Chl *a* measurements, 1 ml of the crushed tissue homogenate was centrifuged at 5,000 × *g* for 10 min at 4°C (Labofuge 400R; Heraeus Instruments) and the supernatant was discarded, leaving the coral pellet. Pigments were extracted from the coral pellet after an incubation in 10 ml of 90% acetone at 4°C during 24 h in the dark, followed by centrifugation at 13,000 × *g* for 10 min (Biofuge Pico; Heraeus Instruments). Absorbance readings at 750, 664, 647, and 630 nm were performed as described previously by Jeffrey and Humphrey (77).

### (i) Coral metagenomes.

Two coral replicates per treatment were used to extract total DNA from the crushed tissue by the use of an UltraClean soil DNA kit (Mo Bio; Carlsbad, CA, USA) following the manufacturer’s instructions for maximum yield. Samples were sequenced using 150-bp single-end reads on an Illumina HiSeq 2000 platform. Raw reads were cleaned up by trimming adaptor sequences and low-quality ends (quality score < 30) using Prinseq (71).

Fragment recruitments were performed for different *Vibrio* species to estimate the abundances of these species in the coral samples. We used a database with a total of 9 *Vibrio* genomes (including V. mediterranei strain Vic-Oc-097 and V. coralliilyticus strain Vic-Oc-068, sequenced in this work, and 7 *Vibrio* spp. previously reported in corals, obtained from GenBank; see Table S1 in the supplemental material). Fragment recruitment analyses were carried out with BLASTN comparisons using version 2.2.31+. Only reads with identities of over 95% and 70% query (read) coverage were considered. When a read hit equally for several vibrios, it was taken into account for all of them using a modification of the BlastTab.best_hit_sorted of Enveomics Toolbox (78). The BlastTab.cast script was used to calculate the sequencing depth of the *Vibrio* genomes and to draw the recruitment plots (enveomics.R library).

10.1128/mBio.00201-20.7 TABLE S1 *Vibrio* genomes used for fragment recruitment analyses. Genomes were obtained from the NCBI Reference Sequence (RefSeq) database of genomes. Download Table S1, DOCX file, 0.01 MB. Copyright © 2020 Rubio-Portillo et al. 2020 Rubio-Portillo et al. This content is distributed under the terms of the Creative Commons Attribution 4.0 International license.

10.1128/mBio.00201-20.8 TABLE S2 Main genome features of V. coralliilyticus strain Vic-Oc-068 and V. mediterranei strain Vic-Oc-097 sequenced in this work. Download Table S2, DOCX file, 0.01 MB. Copyright © 2020 Rubio-Portillo et al. 2020 Rubio-Portillo et al. This content is distributed under the terms of the Creative Commons Attribution 4.0 International license.

10.1128/mBio.00201-20.9 TABLE S3 Transcriptome features. Download Table S3, DOCX file, 0.01 MB. Copyright © 2020 Rubio-Portillo et al. 2020 Rubio-Portillo et al. This content is distributed under the terms of the Creative Commons Attribution 4.0 International license.

10.1128/mBio.00201-20.10 TABLE S4 Comparison of transcriptomic results in V. mediterranei and V. coralliilyticus cells grown in monoculture and coculture conditions at 20 and 28°C. Download Table S4, DOCX file, 0.01 MB. Copyright © 2020 Rubio-Portillo et al. 2020 Rubio-Portillo et al. This content is distributed under the terms of the Creative Commons Attribution 4.0 International license.

### (ii) Coral metabolomes and LC-MS/MS data acquisition.

One coral replicate per treatment was used to analyze the coral metabolomes. Crushed coral tissue was mixed with 5 ml of cold 70% methanol, incubated for 12 h at 4°C, and stored at –80°C until further analysis. Samples were resuspended in 100% methanol containing 2 μM sulfamethazine as an internal standard, and liquid chromatography-tandem mass spectrometry (LC-MS/MS) analysis was performed in an UltiMate 3000 UPLC (ultraperformance liquid chromatography) system (Thermo Scientific) using a Kinetex 1.7-μm C_18_ 100 Å reversed-phase UHPLC (ultra-high-performance liquid chromatography) column (50 by 2.1 mm) and Maxis Q-TOF (quadrupole-time of flight) mass spectrometer (Bruker Daltonics) equipped with an electrospray ionization (ESI) source. The column was equilibrated with 5% solvent B (LC-MS-grade acetonitrile, 0.1% formic acid) for 1 min, followed by a linear gradient from 5% solvent B to 100% solvent B in 8 min and a hold at 100% solvent B for 3 min. Then, the gradient dilution was changed from 100% solvent B to 5% solvent B in 1 min and was maintained at 5% solvent B for 2 min at a flow rate of 0.5 ml/min throughout the run. MS spectra were acquired in positive-ion mode in the range of 100 to 2,000 *m/z*. Sulfamethazine (2 μM), used as an internal standard, was run after 12 samples (after each row in a 96-well plate). A mixture of 10 mg/ml each of sulfamethazine, sulfamethizole, sulfachloropyridazine, sulfadimethoxine, amitriptyline, and coumarin-314 was run before the analysis and after every 48 injections for quality control. An external calibration with ESI–low-concentration tuning mix (Agilent Technologies) was performed prior to data collection, and the internal calibrant Hexakis (1H,1H,2H-perfluoroethoxy) phosphazene (CAS 186817-57-2) was used throughout the runs. A capillary voltage of 4,500 V, a nebulizer gas pressure (nitrogen) of 2 bar, an ion source temperature of 200°C, a dry gas flow of 9 liters/min source temperature, and spectral rates of 3 Hz for MS1 and 10 Hz for MS2 were used. For acquiring MS/MS fragmentation, the 5 most intense ions per MS1 were selected, the MS/MS active exclusion parameter was enabled and set to 2 and to release after 30 s, and each precursor ion was reconsidered for MS/MS if the current intensity/previous intensity ratio was >2.

Feature finding was performed with open source MZmine software (79, 80) version 2.35 using the following settings: mass detection [MS1 detection at noise level of 10^4^, MS2 detection at noise level of 10^2^]; chromatogram building [Min time span: 0.01 min; Min height: 3 × 10^4^; *m/z* tolerance: 25 ppm]; deconvolution [Algorithm: Baseline cut-off (Min Peak height: 10^4^; Peak duration range 0.01–1.0 min; baseline level: 1.0 × 10^2^); *m/z* range for MS2 scan pairing: 0.01 Da; RT range for MS2 scan pairing: 0.3 min]; isotopic peak grouper [*m/z* tolerance: 25 ppm; RT tolerance: 0.2 min; Max charge: 2]; alignment [Join Aligner, *m/z* tolerance: 25 ppm; weight for *m/z*: 75; weight for RT: 25; RT tolerance: 0.2 min]; Gap filling [Peak finder, Intensity tolerance: 1%; *m/z* tolerance: 25 ppm; RT tolerance: 0.2 min; RT correction: checked]; duplicate Peak Filter [Filter mode: new average (create consensus row from duplicates); *m/z* tolerance: 10 ppm; RT tolerance: 0.08 min]; peak filter [Peak area 104–107; Peak duration: 0.00 - 1.00 min]; and peak Row Filtering to export .mgf file to GNPS (Minimum peaks in a row: 2; RT: 1.00-14.00 min). Spectral alignment and molecular networking were performed using the GNPS platform (81, 82). Compounds were identified using GNPS spectral libraries corresponding to level 2 annotations as described previously by Sumner et al. (83). The parameters used for molecular networking analyses can be accessed via the following link: https://gnps.ucsd.edu/ProteoSAFe/status.jsp?task=2bb970e9782f40eaa50d8b375cfb7444.

### Data availability.

V. mediterranei and V. coralliilyticus genomes were published in GenBank-NCBI under the following BioProject numbers: PRJNA632837 and PRJNA632838. The raw sequences of the transcriptome and metagenome data sets have been deposited in NCBI Sequence Read Archive (SRA) database under BioProject accession number PRJNA609971 and BioProject PRJNA612159, respectively. LC-MS/MS data were deposited in the online repository MassIVE under accession number MSV000083610 and can be accessed as “GNPS - Metabolomes from O. patagonica - V. mediterranei - V. coralliilyticus cocultures” via the following link: ftp://massive.ucsd.edu/MSV000083610.
