# Supplementary material for: Virulence as a Side Effect of Interspecies Interaction in Vibrio Coral Pathogens
Source: mBio. 2020 Jul 21;11(4):e00201-20. doi: 10.1128/mBio.00201-20 (PMC7374056; doi:10.1128/mBio.00201-20)
Supplement: TABLE S1 [file mBio.00201-20-st001.docx]

**Table S1**. *Vibrio* genomes used for fragment recruitment analyses. Genomes were obtained from NCBI Reference Sequence (RefSeq) database of genomes.

| *Vibrio* species | Strain | Accession number | Sequence length |
| --- | --- | --- | --- |
| *Vibrio atlantius* | CECT 7223 | GCF_900089855.1 | 4,990,786 |
| *Vibrio breoganii* | FF50 | GCF_001677275.1 | 4,492,180 |
| *Vibrio splendidus* | BST398 | GCF_003345295.1 | 5,508,387 |
| *Vibrio alginolyticus* | ATCC 17749 | GCF_000354175.2 | 5,146,637 |
| *Vibrio harveyi* | ATCC 33843 | GCF_000770115.1 | 5,881,490 |
| *Vibrio tubiashii* | ATCC 19109 | GCF_000772105.1 | 5,540,337 |
| *Vibrio parahaemolyticus* | O3:K6 | GCF_000196095.1 | 5,165,770 |
