# Supplementary material for: Virulence as a Side Effect of Interspecies Interaction in Vibrio Coral Pathogens
Source: mBio. 2020 Jul 21;11(4):e00201-20. doi: 10.1128/mBio.00201-20 (PMC7374056; doi:10.1128/mBio.00201-20)
Supplement: TABLE S2 [file mBio.00201-20-st002.docx]

**Table S2**. Main genome features of the of *V. coralliilyticus* strain Vic-Oc-068 and *V. mediterranei* strain Vic-Oc-097 sequenced in this work.

|  | ***V. coralliilyticus* Vic-Oc-068** | | | | ***V. mediterranei* Vic-Oc-097** | | |
| --- | --- | --- | --- | --- | --- | --- | --- |
|  | (ANIb of 97.22% to *V. coralliilyticus* RE22) | | | | (ANIb of 98.76% to *V. shilonii AK1*) | | |
|  | **Chr1** | **Chr2** | **Total** | **Chr1** | | **Chr2** | **Total** |
| **Size (bp)** | 3,443,835 | 1,985,733 | 5,429,568 | 3,644,912 | | 2,006,290 | 5,651,202 |
| **Contigs** | 12 | 2 | 14 | 7 | | 1 | 8 |
| **GC content (%)** | 45.6 | 45.3 | 45.5 | 44.3 | | 43.7 | 44.1 |
| **ORFs** | 3126 | 1786 | 4912 | 3305 | | 1798 | 5103 |
|  |  |  |  |  |  |  |  |
| **Annotated ORFs** | 2,846 | 1,515 | 4,361 | 3,022 | | 1,582 | 4,604 |
| **ORFs with conserved unknown function** | 98 | 108 | 206 | 133 | | 96 | 229 |
| **ORFs coding for hypothetical proteins** | 280 | 271 | 551 | 283 | | 216 | 499 |
|  |  |  |  |  |  |  |  |
| **tRNAs** | 84 | 5 | 89 | 78 | | 6 | 84 |
| **rRNA operons** | 7 | 0 | 7 | 5 | | 0 | 5 |
