# Supplementary material for: Virulence as a Side Effect of Interspecies Interaction in Vibrio Coral Pathogens
Source: mBio. 2020 Jul 21;11(4):e00201-20. doi: 10.1128/mBio.00201-20 (PMC7374056; doi:10.1128/mBio.00201-20)
Supplement: TABLE S3 [file mBio.00201-20-st003.docx]

**Table S3.** Transcriptomes genomic features.

|  | Number of seqs | Ave. Length (pb) | seqs 16S rRNA & 23S rRNA | % rRNA | Mb mRNA |
| --- | --- | --- | --- | --- | --- |
| 20M1_R1_deduplicate | 1662164 | 127 | 1212180 | 27.86 | 58.80 |
| 20M2_R1_deduplicate | 2146226 | 130.1 | 1448903 | 33.13 | 92.50 |
| 20MIX1_R1_deduplicate | 9075879 | 120 | 5747153 | 36.06 | 392.90 |
| 20MIX2_R1_deduplicate | 8976076 | 100.4 | 5136494 | 41.63 | 375.25 |
| 20C1_R1_deduplicate | 4194720 | 129 | 2774669 | 34.36 | 185.98 |
| 20C2_R1_deduplicate | 6062807 | 115.3 | 3578137 | 40.64 | 284.15 |
| 28M1_R1_deduplicate | 5523110 | 117.4 | 3324504 | 39.48 | 255.93 |
| 28M2_R1_deduplicate | 5100247 | 119.4 | 3179940 | 37.34 | 227.38 |
| 28C1_R1_deduplicate | 3042687 | 122.4 | 1784855 | 41.72 | 155.33 |
| 28C2_R1_deduplicate | 2178544 | 137.8 | 1301593 | 40.84 | 122.57 |
| 28MIX1_R1_deduplicate | 1289915 | 140.2 | 786451 | 39.88 | 72.14 |
| 28MIX2_R1_deduplicate | 1881710 | 135.5 | 1161174 | 39.19 | 99.90 |
