# Supplementary material for: Virulence as a Side Effect of Interspecies Interaction in Vibrio Coral Pathogens
Source: mBio. 2020 Jul 21;11(4):e00201-20. doi: 10.1128/mBio.00201-20 (PMC7374056; doi:10.1128/mBio.00201-20)
Supplement: TABLE S4 [file mBio.00201-20-st004.docx]

**Table S4**. Comparison of transcriptomic results in V. mediterranei (Vm) and V. coralliilyticus (Vc) cells grown in monoculture and coculture at 20 and 28ºC.

| **Strains** | ***V. coralliilyticus* Vic-Oc-068** | | | | ***V. mediterranei* Vic-Oc-097** | | | |
| --- | --- | --- | --- | --- | --- | --- | --- | --- |
| **Growth conditions (ºC)** | 20 | 28 | Mix20 | Mix28 | 20 | 28 | Mix20 | Mix28 |
| Percentage of expressed ORFs | 72.71 ± 1.23 | 82.34 ± 4.53 | 53.18 ± 4.01 | 79.26 ± 1.45 | 67.74 ± 2.98 | 84.55 ± 6.97 | 82.01 ± 1.40 | 64.98 ± 2.10 |
| Chromosome I | 74.01 ± 1.46 | 81.99 ± 9.26 | 54.24 ± 5.22 | 80.29 ± 1.53 | 72.89 ± 3.59 | 88.30 ± 8.63 | 86.70 ± 2.15 | 69.88 ± 5.78 |
| Chromosome II | 68.44 ± 2.07 | 77.97 ± 4.50 | 45.71 ± 5.90 | 77.35 ± 0.89 | 65.66 ± 5.77 | 86.64 ± 12.99 | 82.17 ± 1.88 | 63.04 ± 5.92 |
